# A Rapid and Highly Sensitive Method of Non Radioactive Colorimetric *In Situ* Hybridization for the Detection of mRNA on Tissue Sections

**DOI:** 10.1371/journal.pone.0033898

**Published:** 2012-03-30

**Authors:** Electra Stylianopoulou, Dimitrios Lykidis, Petros Ypsilantis, Constantinos Simopoulos, George Skavdis, Maria Grigoriou

**Affiliations:** 1 Department of Molecular Biology and Genetics, Democritus University of Thrace, Alexandroupolis, Greece; 2 School of Medicine, Democritus University of Thrace, Alexandroupolis, Greece; University of Giessen Lung Center, Germany

## Abstract

**Background:**

Non Radioactive colorimetric *In Situ* Hybridization (NoRISH) with hapten labeled probes has been widely used for the study of gene expression in development, homeostasis and disease. However, improvement in the sensitivity of the method is still needed to allow for the analysis of genes expressed at low levels.

**Methodology/Principal Findings:**

A stable, non-toxic, zinc-based fixative was tested in NoRISH experiments on sections of mouse embryos using four probes (*Lhx6, Lhx7, ncapg and ret*) that have different spatial patterns and expression levels. We showed that Z7 can successfully replace paraformaldehyde used so far for tissue fixation in NoRISH; the morphology of the cryosections of Z7-fixed tissues was excellent, and the fixation time required for tissues sized 1 cm was 1 hr instead of 24 hr for paraformaldehyde. The hybridization signal on the sections of the Z7-treated embryos always appeared earlier than that of the PFA-fixed embryos. In addition, a 50–60% shorter detection time was observed in specimen of Z7-treated embryos, reducing significantly the time required to complete the method. Finally and most importantly, the strength of the hybridization signal on the sections of the Z7-treated embryos always compared favorably to that of the sections of PFA-fixed embryos; these data demonstrate a significant improvement of the sensitivity the method that allows for the analysis of mRNAs that are barely or not detected by the standard colorimetric NoRISH method.

**Conclusions/Significance:**

Our NoRISH method provides excellent preservation of tissue morphology, is rapid, highly sensitive, and especially suitable to implement in the study of genes expressed at low levels and/or in sparse cells within a structure.

## Introduction

*In situ* hybridization (ISH) is a powerful method for the spatiotemporal analysis of gene expression and provides valuable insights into the understanding of the molecular mechanisms implicated in development, homeostasis and disease (see [1] for a recent review). ISH is based on the use of an RNA probe to detect, usually on tissue sections, the presence of complementary mRNA sequences. Depending on the label of the probe, two types of ISH have been developed: the radioactive ISH (RISH) and the non-radioactive ISH (NoRISH). RISH has the advantages of being both quantitative and very sensitive. However, it is a time-consuming method because of the long exposure time – sometimes up to one month – required for the detection of the radioactive probe [2]–[17]. Moreover, since the signal is detected in the emulsion which covers the section, in a different plane of focus than the target sequence, the quality of the results is compromised. On the other hand, NoRISH with hapten labeled probes such as digoxigenin, is a robust, safe and efficient method with very good sensitivity. Colorimetric NoRISH has been widely used for many years since it allows for the analysis of gene expression up to the single-cell level and is particularly useful for the study of complex tissues and organs with non uniform structure such as the brain [1], [11], [13], [18]–[21]. Nevertheless, as NoRISH is not as sensitive as RISH, the detection of low levels of gene expression and/or expression in few cells within a structure is difficult. To overcome this problem an amplification step, usually using tyramide-based commercial kits, has been introduced [22]–[24]. However, this amplification step reduces signal-to-noise ratio and therefore, in order to obtain meaningful results, careful adaptation of the method is required. Furthermore, tyramide-based amplification increases the cost by 50–70%; these expenses may reach an annual cost of few thousand euros.

The sensitivity of NoRISH depends mainly on the quality of mRNA, its retention in the tissue, as well as the accessibility of the target sequences to the probe; these factors are directly influenced by the treatment of the specimen prior to hybridization [1], [7], [10], [15], [25]–[29]. For optimum RNA integrity and quality tissues are fresh frozen [1], [13], [30]–[37]. Yet, as the interpretation of the results of ISH is based on the evaluation of the signal within the tissue, excellent preservation of the structure is also required, thus the specimen is usually fixed. Fixation however, is known to affect RNA quality and quantity and to hamper the penetration of the probe reducing hybridization sensitivity and efficiency [1], [25], [26], [29], [38]. So far the fixative of choice for NoRISH has been paraformaldehyde (PFA) which provides a reasonable compromise between integrity and retention of the RNA, accessibility to the probe and preservation of the morphology [1], [13], [15], [25]–[27], [29], [38]. Zinc-based fixation, although first described in 1994 [35] as an alternative to formalin fixation for paraffin embedded sections, has not been used extensively so far [34], [35]–[37]. In a recent study, 25 zinc-based fixatives were compared to neutral buffered formalin (NBF) by assessing the quality/quantity of nucleic acids and proteins from paraffin- embedded tissues [34]. One of these fixatives, Z7 (zinc acetate, zinc chloride and calcium chloride in Tris buffer), was shown to be particularly efficient in preserving RNA quantity as well as RNA integrity [34]. Interestingly, the quality of the RNA isolated from Z7-fixed specimen was nearly as good as that of the RNA isolated from snap-frozen samples [34]. For the methods used to detect mRNA transcripts, such as NoRISH, significant improvement in the preservation of the RNA quality and quantity in the target tissue, is associated with an increase of the sensitivity of the method. Therefore, we sought to investigate whether Z7 fixative could be used to improve the sensitivity of the NoRISH method. To this end we performed NoRISH experiments on sections of mouse embryos using four probes that have different spatial patterns and expression levels [39], [40]. We show that Z7 can successfully replace PFA; the morphology of Z7-fixed tissues is excellent and the fixation time required is reduced to 1 hr instead of 24 hr for the standard protocol. In addition the detection time of the colorimetric reaction is drastically reduced by 60%. Finally and most importantly, the introduction of Z7 improves significantly the sensitivity of the method allowing for the analysis of mRNAs that are barely or not detected by the standard colorimetric NoRISH method.

## Results

**Figure 1:**
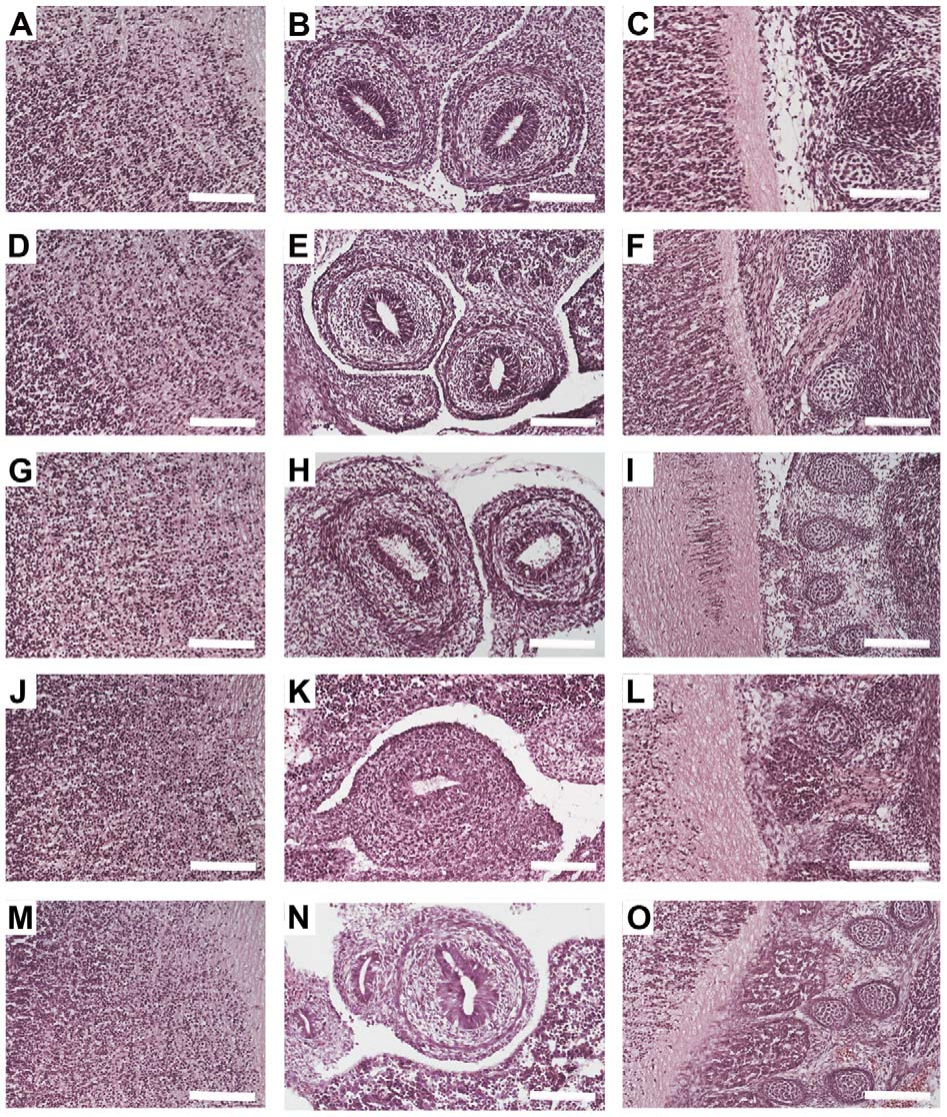
The morphology of cryosections of Z7- fixed tissues is comparable to that of PFA- fixed tissues. Haematoxylin and Eosin Staining (H&E) on saggital sections of E13.5 mouse embryos fixed in Z7 for 1 h (A–C), 3 h (D–F), 6 h (G–I), 24 h (J–L) or in PFA for 24 h (M–O). Three areas are shown: brain (A, D, G, J and M), gut (B, E, H, K and N) and spinal cord/DRGs (C, F, I, L and O). Scale bar: 100 µm.

NoRISH is usually performed on cryosections of tissue specimen, as paraffin embedding has been shown to hamper the detection of mRNA transcripts [8]–[15]. Yet, all the studies of the mophology that have been performed so far using zinc fixation have been done on paraffin embedded tissues [34], [35]–[37], [42]. Therefore in order to investigate whether Z7 can be used to improve the sensitivity of NoRISH we had first to study the morphology of the tissues on cryosections of Z7-fixed embryos and compare it to that of PFA-fixed embryos. To this end cryosections of E13.5 C57/BL6 littermate mouse embryos fixed either in PFA (24 h – our standard protocol) or in Z7 (24 h), were prepared and histological examination of several embryonic tissues and organs was performed following H&E staining. The morphology of Z7-fixed tissues was comparable to that of PFA-fixed tissues (Table 1). Examples of typical morphology of three different areas of the embryo are shown in Figure 1 (J-O). We did, however, notice that despite the very good morphology of the tissues, Z7 fixation caused a slight shrinkage to the specimen. As this could be the due to overfixation of the tissues we decided to test Z7 for shorter fixation time. Cryosections of E13.5 C57/BL6 littermate mouse embryos fixed either in PFA (24 h) or in Z7 (1 h, 3 h, 6 h, 12 h) were prepared and histological examination of several embryonic tissues and organs was performed following H&E staining. Surprisingly, as little as 1 h fixation in Z7 was enough to preserve the morphology of the embryonic tissues (Fig. 1 A–I, Table 1), while at the same time no reduction in the size of the embryo was observed.

**Table 1:** The morphology of Z7-fixed tissues is comparable to that of PFA-fixed tissues.

Embryonic Tissue	Nuclear details	Cytoplasmic details	Cell membrane details
**PFA 24 h**			
Brain	3	3/4	4
Liver	4	3/4	4
Limb	4	3	4
Heart	3	4	4
**Z7 24 h**			
Brain	2/3	3	3/4
Liver	3	3	3/4
Limb	3/4	3	3/4
Heart	3	4	3/4
**Z7 12 h**			
Brain	2/3	3/4	3/4
Liver	3/4	3/4	3/4
Limb	4	3	4
Heart	3	4	4
**Z7 6 h**			
Brain	3	3/4	4
Liver	4	3/4	4
Limb	4	3	4
Heart	3	4	4
**Z7 3 h**			
Brain	3	3/4	4
Liver	4	3/4	4
Limb	4	3	4
Heart	3	4	4
**Z7 1 h**			
Brain	3	3/4	4
Liver	4	3/4	4
Limb	4	3	4
Heart	3	4	4

Histological assessment of the morphology of the sections of Z7-fixed and PFA-fixed embryos following H&E staining. Tissue morphology was evaluated on a four-point scale, with 1 being poor, and 4 being excellent.

**Figure 2:**
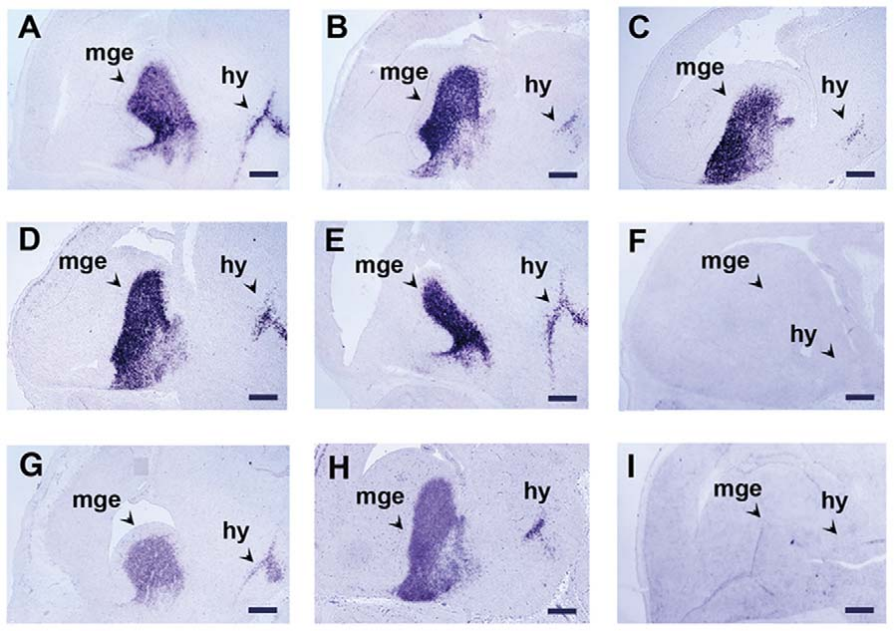
The introduction of Z7 improves the sensitivity of NoRISH. *In situ* hybridization on sections of E13.5 mouse embryo head fixed with Z7 (A–F) or PFA (G–I) and hybridized with a sense (F, I) or an antisense RNA probe of the *Lhx6* gene (A–E, G,H). A: Z7 1 h, B: Z7 3 h, C: Z7 6 h, D: Z7 12 h, E: Z7 24 h, F: Z7 1 h, G–I: PFA 24 h. Detection time: 3 h (A–F), 6 h (G–I). mge: medial ganglionic eminence, hy: hypothalamus. Scale bar: 250 µm.

Having established that the quality of the Z7-fixed tissues was very good, we then hybridized cryosections of the Z7-fixed and the PFA-fixed (control) embryos with an antisense probe for the *Lhx6* gene which encodes for a transcription factor expressed in the developing brain and branchial arches [39]. Typical results of these experiments are presented in Figure 2; in the brain, *Lhx6* mRNA was detected in the medial ganglionic eminences of the telencephalon as well as in the hypothalamus. The preservation of the tissue morphology was very good and allowed clear observation of the cellular localization of the signal. Moreover, the strength of the hybridization signal on the sections of the Z7-treated embryos- irrespectively of the fixation time as well as at the end of the detection- compared favorably with that of the PFA-fixed embryos (Fig. 2A–2E, 2G, 2H). In addition, the signal appeared much earlier on the sections of Z7-fixed embryos (30–60 min) than on the sections of PFA-fixed embryos (2 h – Table 2). These results showed that Z7 can successfully replace PFA and suggested that fixation with Z7 improves the sensitivity of the method. Yet, as the improvement in the sensitivity could compromise specificity we also hybridized cryosections of Z7-fixed and PFA-fixed embryos with the sense probe for the *Lhx6* gene (Fig. 2F, 2I). These experiments showed that the introduction of Z7 fixative did not affect the specificity of the method.

**Table 2:** Tissue fixation in Z7 improves the sensitivity of NoRISH.

Tissue fixation (fixative, time)	Probe	Appearance of the signal (h)	Detection time (h)	Signal intensity
PFA 24 h	*Lhx6*	2 h	6	3
Z7 1 h	*Lhx6*	1/2–1 h	3	4
PFA 24 h	*Lhx7*	2 h	6	3
Z7 1 h	*Lhx7*	1/2–1 h	3	4
PFA 24 h	*ret*	3 h	10	3
Z7 1 h	*ret*	1 h	3	4
PFA 24 h	*ncapg*	10–12 h	36–48	1–2*
Z7 1 h	*ncapg*	3 h	10–12	4

Summary of the results of *in situ* hybridization experiments on sections of mouse embryos with *Lhx6*, *Lhx7*, *ret* and *ncapg* antisense probes. Signal intensity was evaluated on a four-point scale, with 1 being weak, and 4 being very strong. -: no signal.

* depending on the stage.

We then extended our study and analyzed, the expression of three other genes -namely *ncapg* (encodes for the non-SMC condensin I complex subunit G which is probably implicated in cell division), *Lhx7* (encodes for a transcription factor) and *ret* (encodes for a receptor tyrosine kinase) - that have different spatial patterns and expression levels [39]–[Bibr pone.0033898-Pachnis1] [41]. In the mouse embryo *Lhx7* mRNA expression is strong [39], whereas *ret* mRNA expression is moderate [40]. Finally, *ncapg* mRNA expression is at E12.5 very low, close to the detection limit of the method, while at later stages (E13.5-E17.5) is moderate.

**Figure 3:**
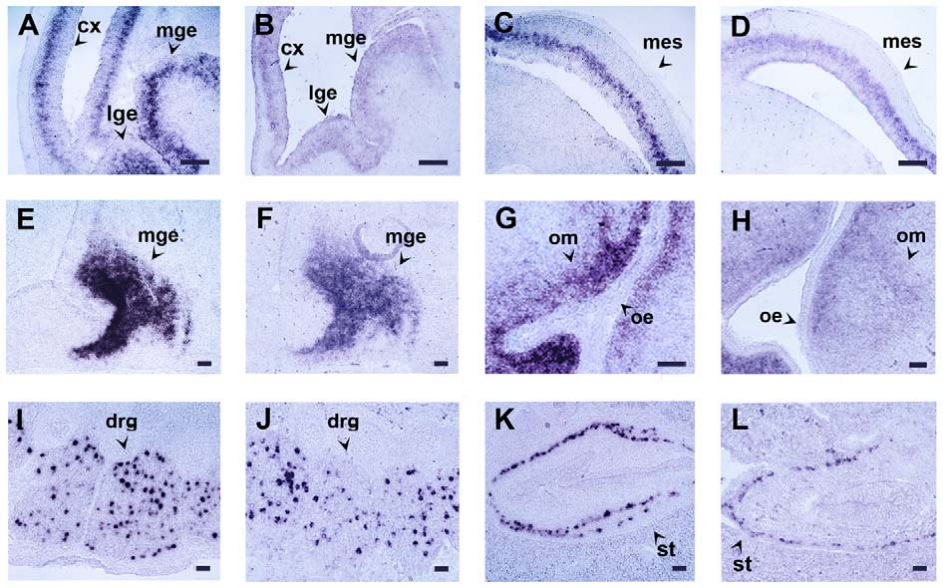
A rapid NoRISH protocol which allows clear detection of the cellular localization of the signal. *In situ* hybridization on sections of E13.5 mouse embryos fixed with Z7 for 1 h (A, C, E, G, I, K) or PFA for 24 h (B, D, F, H, J, L) and hybridized with antisense RNA probes against three different genes. *ncapg* (A–D), *Lhx7* (E–H), *ret* (I–L). Detection time: A, C: 10 h - B, D: 36 h - E, G: 3 h - F, H: 6 h - I, K: 3 h - J, L: 10 h. cx: cortex, drg: dorsal root ganglion, mge: medial ganglionic eminence, mes: mesencephalon, lge: lateral ganglionic eminence, oe: oral epithelium, om: oral mesenchyme, st: stomach. Scale bar: 100 µm.

**Figure 4:**
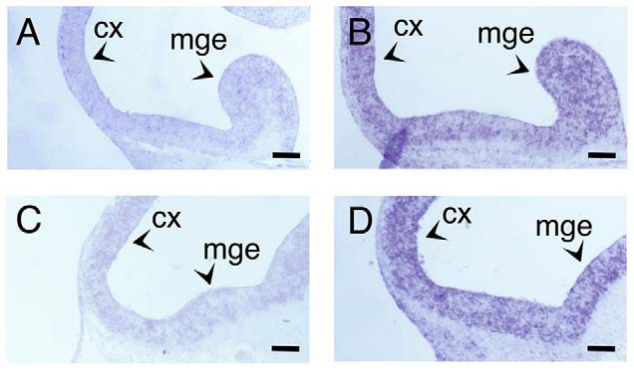
A NoRISH protocol that enables the unequivocal detection of rare mRNA transcripts. *In situ* hybridization on sections of E12.5 mouse embryo head fixed with Z7 (B, D) or PFA (A, C) and hybridized with antisense RNA probe against the *ncapg* gene. Detection time: A, C: 48 h, B: 7 h, D: 12 h. mge: medial ganglionic eminence, cx: cortex. Scale bar: 100 µm.

The results of the *in situ* hybridization experiments performed with the antisense probes against *ncapg*, *Lhx7* and *ret* are presented in Table 2, Figure 3, Figure 4 and Figure S1. At E13.5 *Lhx7* expression appeared in cell populations of the developing telencephalon and oral mesenchyme. On the other hand, at this stage, *ret* expression was restricted in subsets of neuronal cells in the developing brain (not shown), the dorsal root ganglia and the gut. Finally, at this stage *ncapg* was expressed in the ventricular zone of the developing brain. As in the case of *Lhx6*, the signal of *Lhx7*, *ret* and *ncapg* appeared earlier on the Z7-fixed tissues (Table 3). Moreover, at any specific time point during the linear phase of the colorimetric detection period, as well as at the end of the detection, the signal on the Z7-fixed sections was always stronger than that of the PFA-fixed tissues (Fig. 3). Compared to PFA-fixed tissues the detection time for Z7-fixed tissues was reduced by approximately 60% (Table 2). For example, the time required to detect the *ncapg* probe on the sections of the Z7-fixed embryos (between 10 and 12 h; Fig. 3 A, C) was considerably less than the time required for the detection of the same probe on the sections of the PFA-fixed embryos (between 36 and 48 h; Fig. 3 B, D). Similar results were obtained for *Lhx7* (3 h detection time for the sections of the Z7-fixed embryos, 6 h for the sections of the PFA-fixed embryos - compare Fig. 3E, G with 3F, H) and *ret* (3 h detection time for the sections of the Z7-fixed embryos, 10 h for the sections of the PFA-fixed embryos - compare Fig. 3 I, K with 3 J, L). Control experiments with the sense probes against *ncapg*, *Lhx7* and *ret* showed that the improvement of the sensitivity of the method did not compromise its specificity.

**Table 3:** A PFA fixation step is an essential requirement for successful NoRISH.

Tissue fixation	Post fixation	Extra fixation step	Signal intensity
(fixative, time)	(fixative, time)	(fixative, time)	
Z7 1 h	Z7 10 min	-	-
Z7 1 h	Z7 20 min	-	-
Z7 1 h	Z7 1 h	-	-
Z7 1 h	Z7 2 h	-	-
Z7 1 h	PFA 10 min	-	4
PFA 24 h	Z7 10 min	-	3
PFA 24 h	PFA 10 min	-	3
Z7 1 h	Acetone 20 min	-	-
Z7 1 h	70% ethanol 10 min	-	-
Z7 1 h	Methanol 10 min	-	-
Z7 1 h	Z7 10 min	PFA 10 min	4
Z7 1 h	PFA 10 min	Z7 10 min	4
Z7 1 h	-	-	-
PFA 24 h	-	-	-

Summary of the results of *in situ* hybridization experiments on sections of E13.5 mouse embryos with the *Lhx6* probe using several combinations of fixatives.

Signal intensity was evaluated on a four-point scale, with 1 being weak, and 4 being very strong. -: no signal.

The improvement of the sensitivity of our method was further assessed by performing *in situ* hybridization experiments on cryosections of Z7-fixed or PFA-fixed E12.5 embryos, with the antisense probe against *ncapg*. As it has been already mentioned *ncapg* expression is, at this stage, very low. Typical results are presented in Figure 4. The signal on the sections of PFA-fixed embryos was weak, very close to the background (Fig. 4A, C). However, on the sections of the Z7-fixed embryos, the signal obtained was much stronger allowing for the unequivocal detection of the *ncapg* mRNA transcripts (compare Fig. 4A,C to 4 B,D). In addition in these experiments the reduction of the time required for the method was striking – the experiments on Z7-fixed tissues were completed in 4 days while the experiments on PFA-fixed embryos required 9 days (reduction by approximately 60%).

In the NoRISH protocol prior to the acetylation step, a short post-fixation step is performed to fix the sections on the slides (see Materials and Methods). We then tested whether Z7 can also be used for this step, thus eliminating the use of PFA which is an unstable and toxic chemical. Cryosections of E13.5 C57/BL6 littermate mouse embryos fixed in Z7 (1 h, 3 h) were post-fixed in Z7 (5 min, 10 min, 20 min, 1 h, 2 h) prior to hybridization with the antisense riboprobe against *Lhx6* (Table 3). No signal was observed even after long detection times (5 days – Table 3). In parallel two criss-cross control experiments were conducted in which the fixation steps were performed as follows: i) embryo fixation in PFA and post-fixation in Z7 or ii) embryo fixation in Z7 and post-fixation in PFA (Table 3). These experiments were successful. However, when Z7 was used for embryo fixation, the signal obtained was significantly higher, in accordance with the results presented above (Fig. 2, 3 and Tables 2, 3). We also examined whether alternative fixatives (methanol, acetone etc.; see Table 3) could be used for post-fixation instead of PFA. These experiments showed that none of these fixatives could replace PFA (Table 3). We also conducted experiments in which Z7 was used both for embryo fixation and post-fixation, and an extra PFA fixation step was added either before or after the post-fixation. These experiments had successful outcome and established that at least for the NoRISH method, a PFA fixation step is an essential requirement (Table 3).

## Discussion

NoRISH with hapten-labeled probes has been widely used for many years for the study of gene expression in development, homeostasis and disease. However, an increase in the sensitivity of the method is still needed to allow for the detection of rare transcripts and/or restricted expression of a gene in few cells within a tissue. In the present study we describe a modified colorimetric NoRISH method that is highly sensitive, rapid, gives excellent tissue morphology and is suitable for the analysis of rare transcripts.

Zinc-based fixatives have been developed as alternative fixatives to formalin for paraffin embedded sections [34], [35]–[37]. Despite the fact that zinc based fixatives have not been extensively used, in the past few years, they have received attention as it has been shown that for paraffin embedded tissues the preservation of RNA, DNA, and protein is better in zinc based fixatives than in NBF [34], [36], [37], [42], [43]. Z7 is a zinc based fixative which, was shown to be particularly efficient in preserving RNA [34]. For NoRISH, which is a method that is used to detect RNA, significant improvement in the quality and/or in the retention of the target RNA within the tissue can enhance the sensitivity of the method. Therefore, we exploited whether this fixative could be used to improve the sensitivity of the NoRISH method. We showed that Z7 can successfully replace PFA in tissue fixation for NoRISH; the morphology of the cryosections of Z7-fixed tissues was excellent, comparable to that of PFA and, following hybridization, the cellular localization of the signal was clearly observed. Furthermore, Z7 is a powerful fixative; the minimum fixation time for tissues sized 1 cm, was 1 hr instead of 24 hr required for fixing in PFA. Given the short fixation time needed when Z7 is applied, fixation and cryoprotection of the specimen can easily be performed in one working day, thus the time required to complete the method is reduced by 24 h. Moreover, the reduction in the fixation time, abolished the shrinkage of the tissues that we and others observed for zinc based fixatives [34], [43]. Finally, compared to PFA, Z7 is much easier to prepare, it is non-toxic and can be stored at room temperature for at least one year. Yet the most important advantage of our modified NoRISH method is that it is characterized by high sensitivity. In our experiments we used NoRISH to detect the expression of four genes (*Lhx6*, *Lhx7*, *ncapg* and *ret*) with different spatial patterns and expression levels [39], [40]. In all cases the signal observed was stronger and the detection time required to identify unequivocally the transcripts was reduced significantly rendering our method very rapid. For example, the transcripts of *ncapg* are barely detected at E12.5 with the standard protocol; the detection time required is 48 h, and the signal obtain very weak. In our modified protocol *ncapg* transcripts at E12.5 are easily identified following a 10–12 h detection period. In addition, our method is highly specific as the enhancement of the sensitivity is due to the better preservation of the target RNA and, as a result better hybridization. Clearly, the improved sensitivity enables the detection of the expression of genes that are transcribed at low levels and/or transcripts that are present in sparse cells within a structure and are barely or not at all detected by the standard NoRISH. It has been shown that the quality of RNA in zinc-fixed tissues is better than in tissues fixed in PFA or in other fixatives; in fact it is similar to that of fresh-frozen tissues [34], [36], [37]. We therefore believe that the high sensitivity that we observed in our experiments with Z7 is the result of the improved quality of the target mRNA sequences. The results of the criss-cross experiments using Z7 and PFA in each of the two fixation steps of the NoRISH method support this conclusion; when Z7 was applied as the tissue fixative the signal obtained was much stronger. The biochemical mechanism by which zinc interacts with proteins and nucleic acids is unknown, but it has been suggested that the zinc and acetate ions introduce structural changes probably by forming ionic chemical bonds with specific amino acid residues [34], [43]. On the other hand, PFA, an aldehyde fixative, forms both intra- and intermolecular cross-links with protein molecules and rigid heteropolymers that retain the morphology of the tissue [26]–[29], [38]. Nevertheless, for a successful outcome of NoRISH one of the two fixation steps has to be performed in PFA; no other fixative or combinations of commonly used fixatives could substitute for this. Interestingly, if Z7 was used for both fixation steps, the experiment was successful only by the addition of an extra fixation step with PFA. We therefore believe that PFA-mediated cross-linking is necessary for the retention of the target sequences in the tissue - without it the RNA is probably washed away during the lengthy procedure that follows.

To summarize, our NoRISH method is characterized by high sensitivity, excellent tissue morphology, is time-efficient and especially suitable for the study of genes with low expression levels and/or expression in sparse cells within a structure.

## Materials and Methods

### Animals

All experiments were conducted in accordance with the European Communities Council Directive of 24 November 1986 (86/609/EEC) under the permit Τ/1571/13.5.09. The protocols were approved by the committee for the Care and Use of Laboratory animals of the Perfecture of Evros, Thrace, Greece.

### Riboprobes

Antisense RNA probes were synthesized by *in vitro* transcription with T3 or T7 RNA polymerase (Takara), according to manufacturer's instructions, using Digoxigenin-11-UTP (Roche). For *Lhx6*, a 410 bp fragment (nt 699–1110 of the mouse cDNA) was used to generate the sense and the antisense probe [39] For *Lhx7*, an 163 bp fragment (nt 1022–1185 of the mouse cDNA) was used to generate the sense and the antisense probe [39]. For *ncapg*, an 820 bp fragment (nt 1832–2652 of the mouse cDNA NM_019438.1) was used to generate the sense and the antisense probe. For *ret*, a 420 bp was used (nt 3956–4376 of the mouse cDNA) to generate the sense and the antisense probe [40].

### Non radioactive in situ hybridization on mouse embryo tissue sections

Time mated C57/BL6 pregnant female mice were euthanized at E12.5 or E13.5 (the day of vaginal plug detection was considered as day 0.5) and embryos were dissected free of maternal tissues in cold phosphate-buffered saline (PBS, pH 7.4). Embryos were then fixed either in 4% w/v PFA for 24 h at 4°C or in Z7 (0.5% w/v zinc chloride, 0.5% w/v zinc trifluoroacetate, 0.05% w/v calcium acetate, 0.1 M Tris–HCl pH:7) [34] for 1 h, 2 h, 3 h, 6 h, 12 h or 24 h at room temperature (RT). Following fixation, the embryos were cryoprotected either in 30% w/v sucrose in PBS (for PFA-fixed embryos) or in 30% w/v sucrose in 0.1 M Tris pH 7.5 (for Z7-fixed embryos), embedded in Tissue freezing medium (Leica Microsystems), sectioned at 12 µm using a cryostat (Leica 1900UV) and transferred to superfrost plus (ROTH) slides. The sections were air-dried for at least 30 min and stored at −80°C until later use.

Sections were post-fixed in 4% w/v PFA in PBS for 10 min or in Z7, washed three times in PBS (or twice in 0.1 M Tris–HCl pH:7, 0.05 M NaCl and once in PBS for Z7) and incubated in acetylating solution (1.3% v/v triethanolamine, 0.03 N HCl, 0.25% v/v acetic anhydrite) for 10 min. Sections were then washed in PBS, incubated in 1% v/v Triton-X-100 in PBS for 30 min and washed three times in PBS. Prehybridization was performed for 4–6 h in buffer H [50% v/v formamide, 5× SSC (0.75 M NaCl, 0.075 M sodium citrate), 5× Denhardt's (0.1% bovine serum albumin, 0.1% and 0.1% Polyvinylpyrrolidone), 250 µg/ml yeast RNA and 500 µg/ml salmon sperm DNA] [41]. Hybridization was performed in a humidified chamber for 16 h at 65°C in H buffer with DIG-labeled probe added (400 µg/ml). Following hybridization sections were sequentially washed in 5× SSC (5 min, 65°C), 0.2× SSC (1 h, 65°C), 0.2× SSC (5 min, RT). Then they were incubated in AB buffer (0.1 M Tris pH 7.5, 0.15 M NaCl) for 5 min, and in blocking solution (10% v/v Foetal Calf Serum in AB) for 1–2 h at RT. Antibody incubation was performed for 16 h at 4°C in AB buffer supplemented with 1% v/v Foetal Calf Serum and anti-DIG antibody coupled to alkaline phosphatase (1∶5000 dilution; Roche). Sections were then washed thoroughly in AB and equilibrated in alkaline phosphatase buffer (AP - 0.1 M Tris–HCl pH: 9.5, 0.1 M NaCl, 0.05 M MgCl_2_) for 5 min. Alkaline phosphatase activity was detected in the dark in AP buffer supplemented with 45 mg/ml 4-nitrobluetetrazolium chloride (NBT, Roche) and 35 mg/ml 5-bromo-4-chloro-3-indolyl-phosphate (BCIP, Roche). The reaction was stopped with PBS and the sections were mounted in Glycergel (Dako). The experiments were repeated three times. Sections were analyzed with an Eclipse E800 microscope (Nikon, Japan) fitted with an Infinity 1 digital camera (Lumenera, Canada). Images were captured using the camera software and assembled in Adobe Photoshop.

### Haematoxylin and Eosin (H&E) staining

Sections were prepared as described for NoRISH. For H&E staining slides were defrosted and sections were post-fixed in 4% w/v PFA for 10 min and then washed in running tap water. Staining with Harry's haematoxylin (Sigma) was performed for 1 min, followed by rinsing in running tap water for approximately 5 min. Sections were then differentiated in 1% v/v HCl in 70% v/v ethanol for 5 sec and washed in running tap water for 3–5 min. Staining with eosin (Sigma) was performed for 3–4 min, followed by rinsing in running tap water for 20–30 sec. Dehydration was performed twice in 95% v/v ethanol and once in absolute ethanol. Finally, sections were cleared in Xylene (Applichem) and mounted in DPX (Sigma). Tissue morphology was evaluated by light microscopy [Eclipse E800 microscope (Nikon, Japan)] and scored on a four-point scale, with 1 being poor, and 4 being excellent. Nuclear, cytoplasmic, and cell membrane details were assessed and given equal weight. Sections were photographed with an Infinity 1 digital camera (Lumenera, Canada). Images were captured using the camera software and assembled in Adobe Photoshop. The experiments were repeated three times and the assessment of the morphology was performed independently by two investigators.

## Supporting Information

Figure S1 **The introduction of Z7 does not affect the specificity of the method.** *In situ* hybridization on sections of E13.5 mouse embryos fixed with Z7 for 1 h (A, C, E) or PFA for 24 h (B,D,F) and hybridized with antisense RNA probes against three different genes. *ncapg* (A,B), *Lhx7* (C,D), *ret* (E,F). Detection time: A: 12 h, B: 48 h, C: 3 h, D: 6 h, E: 3 h, F: 10 h. cx: cortex, drg: dorsal root ganglion, mge: medial ganglionic eminence, Scale bar: 100 µm.
(TIF)
