# Supplementary figures and images for: A Rapid and Highly Sensitive Method of Non Radioactive Colorimetric In Situ Hybridization for the Detection of mRNA on Tissue Sections
Source: PLoS One. 2012 Mar 30;7(3):e33898. doi: 10.1371/journal.pone.0033898 (PMC3316699; doi:10.1371/journal.pone.0033898)

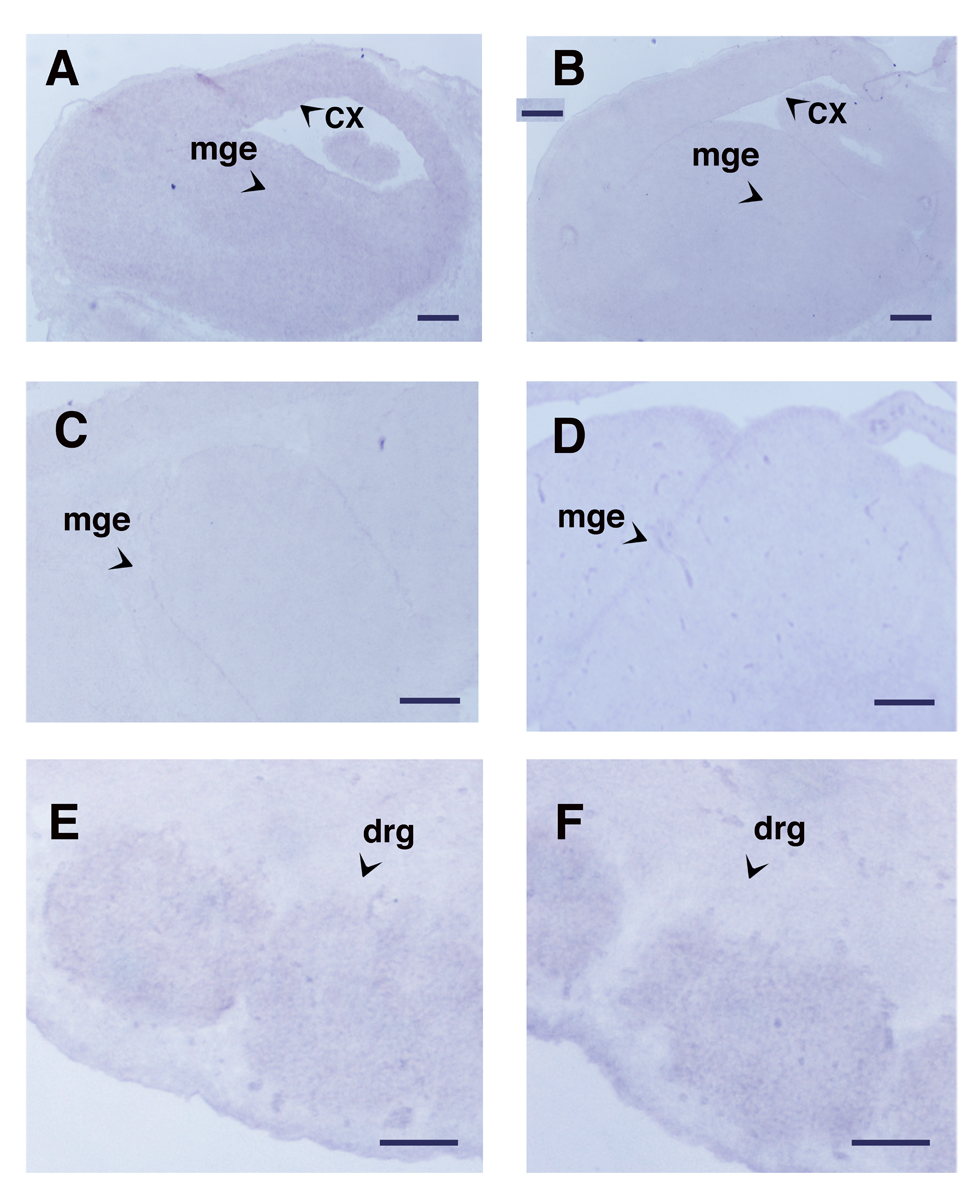

Supplement: Figure S1 — The introduction of Z7 does not affect the specificity of the method. In situ hybridization on sections of E13.5 mouse embryos fixed with Z7 for 1 h (A, C, E) or PFA for 24 h (B,D,F) and hybridized with antisense RNA probes against three different genes. ncapg (A,B), Lhx7 (C,D), ret (E,F). Detection time: A: 12 h, B: 48 h, C: 3 h, D: 6 h, E: 3 h, F: 10 h. cx: cortex, drg: dorsal root ganglion, mge: medial ganglionic eminence, Scale bar: 100 µm. (TIF) [file pone.0033898.s001.tif]
